# Distribution and safety assessment of heavy metals in fresh meat from Zhejiang, China

**DOI:** 10.1038/s41598-022-07214-3

**Published:** 2022-02-25

**Authors:** Jian Long Han, Xiao Dong Pan, Qing Chen

**Affiliations:** Zhejiang Provincial Center for Disease Control and Prevention, Bin Sheng Road No. 3399, Binjiang District, Hangzhou City, 310051 China

**Keywords:** Environmental sciences, Health care

## Abstract

There are increasing concerns on heavy metals in animal derived foods. We analyzed the levels of As, Cd, Cr, Cu, Hg, Ni, and Pb in 1066 fresh meat samples including pork, beef, mutton, chicken and duck from Zhejiang province, southeast China. The average levels of As, Cd, Cr, Cu, Hg, Ni, and Pb were 0.018, 0.002, 0.061, 0.801, 0.0038, 0.055, and 0.029 mg/kg wet weight respectively. There are significant positive correlations among Cd, Hg and Pb (*P* < 0.05) and negative correlations for Cu–Pb or Cu–Cd (*P* < 0.05). The exposure assessment showed that the health risk to humans by consuming these meat products was relatively low. However, regular monitoring of heavy metals in meat products is still recommended considering their intensive industrial activities.

## Introduction

Meat products are major sources of human nutrients, including protein, minerals, vitamins, and fats. Based on Statistical Yearbook of China 2016, Chinese output of pork, beef and mutton reached 86.25 million tones^1^. It has become the main pork producer, accounting for about 50% of the world's production^2^. Although most of Chinese residents have a plant-food-based dietary protein pattern, there is a rapid growth of meat consumption^3^. Recently, concerns have been raised about various toxic elements in meat products.

The contamination of harmful elements in livestock and poultry are caused by animal feeds, especially in some areas with intense manufacturing activities, industrial emissions, coal combustion, and ore mining^4,5^. When toxic elements, such as cadmium (Cd) and lead (Pb) are released into water, soil or air, they could be accumulated by plants and fishes, which are the main raw materials of animal feeds^6–8^. For example, Tao et al.^9^ reported that the incidence rates of cadmium (Cd), mecury (Hg), chromium (Cr), and arsenic (As) contamination for feedstuffs and feeds were high. Furthermore, animal feeds were commonly contaminated with Cr, followed by As, Cd, and Hg. Wang et al.^10^ observed high level of Cr in meat products, which possibly originated from the dietary feeds of animal husbandry.

The high exposure to these metals in meat consumption has negative effects to human body, such as nerve damage, nephropathy and cancers^11–13^. For example, Pb can lead to kidney failure, cardiovascular disease and abnormal nervous development of children^14^. Chronic exposure of Cd can cause liver harm, bone degeneration, blood damage, and renal dysfunction^15^. Hg could damage the nervous system of unborn and newborn children^16^. Therefore, it is necessary to monitor and control toxic elements in meat product from China, one of the largest meat production and consumption countries^17,18^.

Zhejiang province with a high population density is the fast-developing area in the southeast of China. Our previous studies have reported the possible pollution of heavy metals in vegetables, rice, marine fish and seaweeds in Zhejiang^6,11,19–21^. However, to our knowledge, few studies on metal contamination in meat products were reported. The aim of this study was to investigate distribution of heavy metals in livestock and poultry meat and evaluate the health risk to local inhabitants. Our data may provide some insights into toxic elements accumulation in farmed animals and serve as a basis for profiling the public health problem.

## Materials and methods

### Sampling

**Figure 1:**
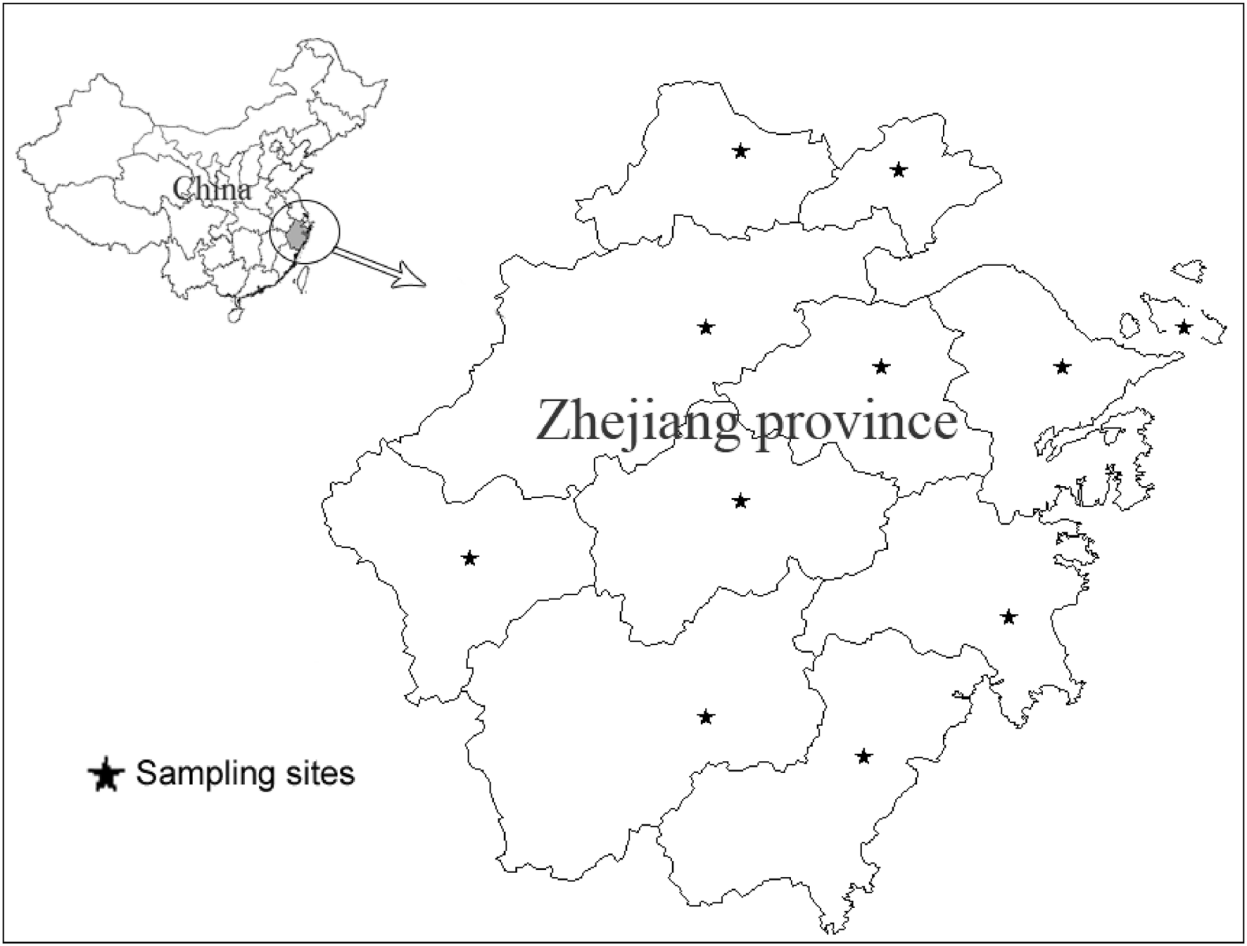
The simple map of sampling areas in Zhejiang province of China.

Total 1066 meat samples were collected in Zhejiang, China whose latitudes range from 27° 09ʹ to 31° 11ʹ N, and the longitudes from 118° 02ʹ to 122° 57ʹ E. Fresh edible meats of livestock and poultry were collected in 11 sampling areas as shown in Fig. 1 which was drawn by software of MapGIS K9 SP2 free trial edition (Zondy Cyber Comp., China, http://www.mapgis.com/index.php/index-view-aid-280.html). The detailed edible parts such as leg, chest and waist were randomly selected. The samples were pork (511), beef (184), mutton (47), chicken (250) and duck (74) collected from 2018 to 2020. All samples transported in plastic bags were refrigerated at − 20 °C until later analysis in the laboratory. The storage period was not more than 7 days.

### Chemical analysis

The concentrations of arsenic (As), cadmium (Cd), chromium (Cr), copper (Cu), mecury (Hg), nickel (Ni), and lead (Pb) were tested according to the Chinese standard analysis method of GB 5009.268–2016^22^. Briefly, samples (0.5–1.0 g) were digested in acid-clean Teflon vessels containing 6 mL HNO_3_ in a Mars-6 microwave digestion system (CEM, Charlotte, NC, USA). The samples in closed vessels were heated at 190 °C for 20 min. After digestion, the residue was heated at 150 °C till nearly dry. For the Hg test, the digested sample was directly diluted without heating for removing residual acid. Then, it was diluted to 20 mL by deionized water for instrumental analysis. As, Cd, Cr, Cu, Hg, Ni, and Pb in all samples were tested using inductively coupled plasma mass spectrometry (ICP-MS) (NexION 300, Perkin Elmer, Inc., Shelton, CT USA). For quality assurance and quality control purposes, sample blanks, certified reference materials (CRMs), and duplicates of the samples (10% of the load) were applied in each batch of treated samples.

### Method validation

The analytical procedures were verified by analysis of appropriate certified reference materials (CRMs) using the same digestion and analytical methods. Two CRMs (Table 1) were purchased from National Research Center for Certified Reference Materials, China (NRCCRM). Quantitative results (no more than 10% of the certified value) were obtained for targeted elements of CRMs. Limits of detection (LODs) were defined as 3 times the standard deviation of 10 runs of blank measurements. LODs of As, Cd, Cr, Cu, Hg, Ni, and Pb were 0.003, 0.001, 0.005, 0.005, 0.0003, 0.004 and 0.004 mg/kg, respectively.

**Table 1:** Determination of certified reference materials (*n* = 6).

	GBW10018 Chicken		GBW10051 pork liver	
	Certified (mg/kg)	Measured (mg/kg)	Certified (mg/kg)	Measured (mg/kg)
As	0.109 ± 0.013	0.099 ± 0.021	1.4 ± 0.3	1.5 ± 0.2
Cd	–	–	1.00 ± 0.07	0.98 ± 0.06
Hg	0.0036 ± 0.0015	0.0039 ± 0.0018	0.045 ± 0.008	0.049 ± 0.011
Pb	0.11 ± 0.02	0.10 ± 0.04	0.12 ± 0.03	0.11 ± 0.06
Cu	1.46 ± 0.12	1.36 ± 0.22	52 ± 3	50 ± 6
Cr	0.59 ± 0.11	0.55 ± 0.17	0.23 ± 0.06	0.19 ± 0.09
Ni	0.15 ± 0.03	0.11 ± 0.08	–	–

### Consumption data

The meat consumption data were provided by the Zhejiang Food and Drug Administration of China^23^. Briefly, it performed the food consumption survey in 2008. In this survey, 9798 people represented certain areas were questioned twice about their last 24-h consumption. Furthermore, the selection of interviewed people and the moment of the interview were designed for a representative consumption profile of the population over 1 year.

### Health risk assessment

According to the recommendation of the report Reliable Evaluation of Low-Level Contaminations of Food issued by WHO, half of LOD was assigned to all results of element levels below the LOD, where the proportion of data below the LOD is not more than 60%^24^.

The targeted hazard quotient (THQ) and hazard index (HI) were used to estimate health risk according to US EPA’s IRIS database^25^. We adopted the mean and 97.5th percentile (P97.5) of obtained element level to represent the consumers with normal and high exposure, respectively^21^. The sum of all THQs for each element was referred as the HI. The formulas were as follows:

1 $${\text{Exposure Dose}} = \frac{Ci \times Di \times Ed}{{Bw \times At}}$$

2 $${\text{Tageted Hazard Quotient }}\left( {{\text{THQ}}} \right) = \frac{{\text{Exposure Dose}}}{RfD}$$

3 $${\text{Hazard Index }}\left( {{\text{HI}}} \right) = \mathop \sum \limits_{k = 1}^{n = k} {\text{Targeted Hazard Quotient}}$$

Ci is the average or P97.5 concentration of the element in meat samples (mg/kg wet weight); Di is the daily intake of livestock and poultry meat (112.9 g/capita/day)^23^; Ed is the average exposure duration (e.g., 70 years)^20^; Bw is the average weight (e.g., 60 kg)^20^; At is the average lifetime (e.g., 70 years)^11^. RfD is the recommended reference dose (RfD)^26^; According to US EPA guidelines for assessing conservative risk, HI were calculated by sum of the THQ. When HI < 1, no health risk is expected to occur; If HI ≥ 1, there is moderate or high risk for adverse human effects.

### Statistical analysis

Data analysis and statistical analysis were performed using Excel (2017 edition) and SPSS16 (Tried edition). The difference was considered as significant by single factor analysis (one-way ANOVA) when *P* < 0.05. The correlation between each factor was analyzed by Pearson correlation analysis.

## Results and discussion

### Heavy metals in meats

Total 1066 meat samples including 511 pork samples, 250 chicken, 184 beef, 74 duck, and 47 mutton purchased from local markets of Zhejiang were analyzed in this study. As shown in Tables 2 and 3, average levels of As, Cd, Cr, Cu, Hg, Ni, and Pb were 0.018, 0.002, 0.061, 0.801, 0.0038, 0.055, and 0.029 mg/kg wet weight respectively. Based on the Chinese National Food Safety standard^27^, the maximum allowable concentrations (MAC) of As, Cd, Cr, Hg and Pb in meat in China were 0.5, 0.1, 1, 0.05 and 0.2 mg/kg. The number of sample exceeding the MAC is 1 for As, 2 for Hg and 10 for Pb. Our results were similar with those found in Beijing China, where there were Cr (0.573 mg/kg), Cd (0.015 mg/kg), Pb (0.167 mg/kg), As (0.053 mg/kg), Hg (0.018 mg/kg) in meats (pork, beef, mutton, chicken)^28^ and the results from Taiwan, China^29^. In some potential polluted areas, average levels of heavy metals, such Cd and Pb were more than 0.2 mg/kg in meat product^30,31^. It shows that levels of these metals in animals change with different area, where may have diverse sources of the contaminant.

**Table 2:** The concentration of heavy metals in meat samples from Zhejiang province (mg/kg fresh weight).

Element	*n*	Mean^a^	P97.5^a^	Range	MAC^b^	No. of > MAC	LOD
As	1066	0.018	0.11	3.2	0.5	1	0.003
Cd	1063	0.002	0.013	0.089	0.1	0	0.001
Cr	1066	0.061	0.318	0.996	1	0	0.005
Cu	1062	0.801	4.09	9.81	–	–	0.005
Hg	1066	0.0038	0.0252	0.076	0.05	2	0.0003
Ni	1066	0.055	0.42	1.4	–	–	0.004
Pb	1060	0.029	0.18	0.536	0.2	10	0.004

^a^Target analytes with concentrations lower than LOD were treated as one-half of LOD when calculating the mean values.

^b^Maximum allowable concentrations of contaminants in foods.

**Table 3:** Comparison of different metals in meat with some previous reports.

Meat type	Area	N	Mean level (mg/kg fresh weight)	References
Pork,	Italy (meat products)	100	Cr 0.15–0.23; Cd 0.01–0.03; Hg 0.01–0.02; Cu 1.08–1.21; Pb 0.22–0.38	Barone et al.^30^
	Beijing, China	–	Cr 0.483; Cd 0.003; Pb 0.029; As 0.043; Hg 0.015	Liang et al.^28^
	Zhejiang, China	511	Cr 0.062; Cd 0.002; Pb 0.058; As 0.020; Hg 0.004; Cu 0.633; Ni 0.058	This study
Beef	Beijing, China	–	Cr 0.504; Cd 0.015; Pb 0.201; As 0.077; Hg 0.010	Liang et al.^28^
	Iran	72	Cd 0.028; Cd 0.028; Hg 0.003	Hashemi^33^
	Zhejiang, China	184	Cr 0.062; Cd 0.002; Pb 0.061; As 0.018; Hg 0.004; Cu 0.673; Ni 0.061	This study
Mutton	Beijing, China	–	Cr 0.654; Cd 0.031; Pb 0.128; As 0.008; Hg 0.005	Liang et al., 2019^28^
	Zhejiang, China	47	Cr 0.045; Cd 0.002; Pb 0.061; As 0.008; Hg 0.003; Cu 0.956; Ni 0.061	This study
Chicken	Beijing, China	–	Cr 0.650; Cd 0.031; Pb 0.291; As 0.045; Hg 0.017	Liang et al.^28^
	Guangzhou, China (Drumstick)	30	Cr 0.11; Cd 0.002; Pb 0.073; As 0.029; Cu 0.757; Ni 0.069	Hu et al.^32^
	Pakistan	60	Cd 0.017; Pb 0.16; Ni 0.39	Abbas et al.^29^
	Zhejiang, China	250	Cr 0.060; Cd 0.003; Pb 0.058; As 0.018; Hg 0.004; Cu 0.535; Ni 0.042	This study
Duck	Thailand	90	Pb 3.13 (dry wet); Cd 0.33 (dry wet); Cu 15.28 (dry wet)	Aendo et al.^31^
	Zhejiang, China	74	Cr 0.073; Cd 0.003; Pb 0.058; As 0.014; Hg 0.004; Cu 3.1; Ni 0.047	This study

**Figure 2:**
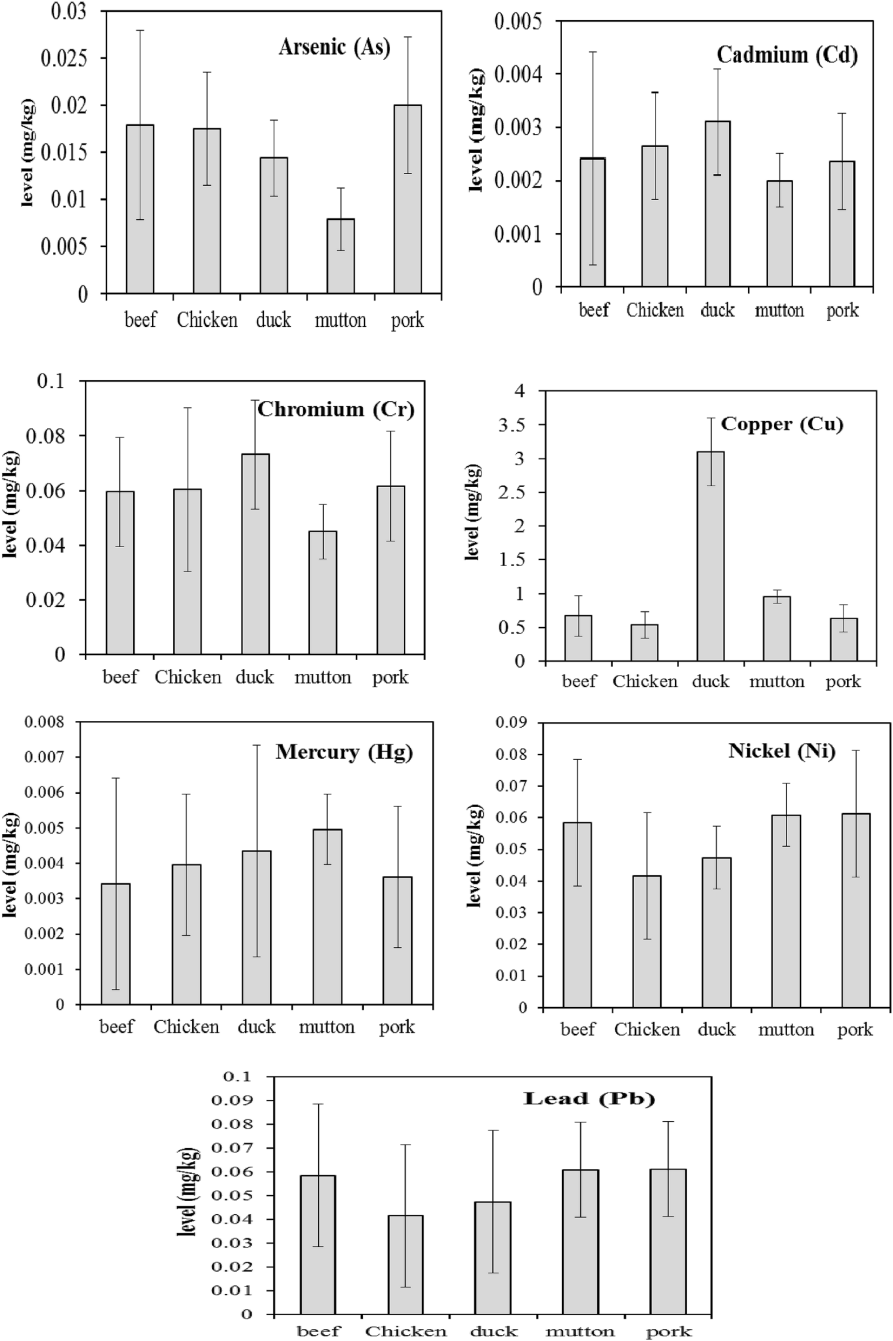
The levels of heavy metals (As, Cd, Cr, Cu, Hg, Ni, and Pb in different meats.

Different animal species may have different bio-accumulation ability to heavy metals. The average levels of As, Cd, Cr, Cu, Hg, Ni, and Pb in different meat samples were shown in Fig. 2. By comparison with beef, chicken, duck and pork, mutton had relative lower levels of As, Cd, and Cr (*P* < 0.05). Mutton accumulated lower levels of As, Cd, and Cr, which may be caused by the grassy feed and less mineral supplement. High Cu concentration (average 3.1 mg/kg) was found in duck meat. Considering the nearly 80% water content in duck meat, our result was similar with the report of Aendo et al.^32^, who found duck meat with 15.28 mg/kg dry weight for Cu in Thailand. For Hg, Ni, and Pb, there was no significant difference among five targeted meats (*P* < 0.05). Furthermore, 4 of 248 chicken muscle samples contained Pb with levels above the safety threshold of 0.2 mg/kg (fresh weight)^27^. The ratio of over-limit was lower than that reported in Guangzhou, China where 2 of 63 muscle samples had Pb contents exceeding this limit^33^. But, the mean level (0.058 mg/kg) was higher than reported in Korea (0.005 mg/kg)^34^. The Cd (0.002 mg/kg) in beef was lower than report of Hashemi^35^ who found 0.28 mg/kg Cd in Iran. Feeds and mineral supplement products may be one of many sources of heavy metal for these animals.

Pearson correlation analysis (Table 4) showed that there were significant positive correlations for Cd-Hg (*r* = 0.9141, *P* < 0.05), Pb-Hg (*r* = 0.98837, *P* < 0.05) and Cd–Pb (*r* = 0.9504, *P* < *0.05*) in meat samples. Negative correlations in Cu–Cd (*r* =  − 0.6515, *P* < 0.05) and Cu-Pb (*r* =  − 0.6101, *P* < 0.05) were found in our results. We suppose that two groups of Cd–Hg–Pb and Cu were accumulated by different sources. Actually, most of livestock and poultry in Zhejiang were farming with artificial feeds which may be the main source of heavy metals. The contamination incidence rates of harmful elements, such as Cd, Hg, Pb, and As in feedstuffs and feeds were high, and the feeds were usually contaminated with contaminated with Cr, followed by As, Cd, and Hg^9^. The mean As contents of chicken feeds collected in Jiangsu province of southern China was reported to be 0.13 mg/kg^36^ while the total contents of As in poultry feeds in northeastern China varied from 0.02 to 6.42 mg/kg^37^. Studies found that level of Cu was 2 to 8 times higher than the required ones in poultry and livestock feeds in China^36,37^. As we known, compounds including Cu element were commonly used as a growth promoter in diets of poultry, especial duck^38^.

**Table 4:** Pearson correlations of the heavy metal pollutants in the meat samples.

	As	Cd	Cr	Cu	Hg	Ni	Pb
As	1						
Cd	0.1039	1					
Cr	0.1096	0.5807	1				
Cu	− 0.0685	− **0.6515**	− 0.3624	1			
Hg	0.1041	**0.9141**	0.5752	− 0.5708	1		
Ni	0.0672	0.5181	0.4162	− 0.2900	0.5052	1	
Pb	0.0981	**0.9504**	0.5695	− **0.6101**	**0.8837**	0.5010	1

Significant values are in [bold].

### Exposure assessment and health risk

According to the data of food consumption survey^23^, the estimated livestock and poultry meat intake was 112.9 g/day/person. The recommended reference doses (RfDs) or safe values were based on previous reports^31,33^. The mean and high exposure was presented by the average and P97.5 elements levels, respectively. As shown in Table 4, mean exposure doses of As, Cd, Cr, Cu, Hg, Ni, and Pb by meat consumption were0.034, 0.004, 0.115, 1.507, 0.007, 0.103, and 0.055 μg/kg bw/day. And high exposure values were 0.207, 0.024, 0.598, 7.696, 0.047, 0.790, and 0.339 μg/kg bw/day. Our mean exposure data (As, Cd, Cr, Hg and Pb) were lower than those reported in Beijing, China^28^.

To appraise the health risk associated with these metals, targeted hazard quotient (THQ) was calculated by dividing daily intake of elements by their reference doses. Hazard index (HI) combined all THQs was adopted to assess the total health risk^39,40^^.^ An HI more than 1 is considered as not safe for human health. As shown in Table 5, all THQs were less than 1. Both mean and P97.5 HIs were no more than 1. HI for P97.5 level presented as the high exposure was 0.768. It indicated that there was low health risk to exposure of common toxic elements by intake of these meats. However, it should be noticed that other potential exposure pathways for foods, such as vegetables, cereals, fruits, and fish might be considered except for livestock and poultry meats.

**Table 5:** Estimated exposure to As, Cd, Cr, Cu, Hg, Ni, and Pb for the general population in livestock and poultry meats from Zhejiang province and the health risk assessment.

Element	Safe value (μg/kg bw/day)	Exposure dose (μg/kg bw/day)		Targeted hazard quotient (THQ)		Hazard index (HI)	
		Mean	P97.5	Mean	P97.5	Mean	P97.5
As	3.0	0.034	0.207	0.011	0.069	0.146	0.896
Cd	0.8	0.004	0.024	0.005	0.031		
Cr	3000	0.115	0.598	0.000	0.000		
Cu	40	1.507	7.696	0.038	0.192		
Hg	0.14	0.007	0.047	0.051	0.339		
Ni	20	0.103	0.790	0.005	0.040		
Pb	1.5	0.055	0.339	0.036	0.226		

## Conclusion

The present study revealed the levels of As, Cd, Cr, Cu, Hg, Ni, and Pb in livestock and poultry meats from Zhejiang of southeast China, which showed samples with 0.09% (As), 0.19% (Hg) and 0.94% (Pb) were exceeding the maximum allowable concentrations set by Chinese legislation. Obvious positive correlations among Cd, Hg and Pb and negative correlations for Cu-Pb and Cu-Cd were found in analyzed samples. Dietary exposure assessment showed that there is relatively low health risk to these elements for general people in Zhejiang province of southeast China. However, it should be noted that the detailed information for animal species, feeding pattern cultivation, and feedstuffs was not involved in this study. Moreover, different heavy metal speciation showed diverse toxicity, such as organic and inorganic mercury. Our future survey will focus on the levels of heavy metal speciation in different animal products and feeds.
